# Angle-dependent rotation velocity consistent with ADP release in bacterial F_**1**_-ATPase

**DOI:** 10.3389/fmolb.2023.1184249

**Published:** 2023-08-02

**Authors:** Nathan Suiter, Sándor Volkán-Kacsó

**Affiliations:** ^1^ Department of Mathematics, Physics and Statistics, Azusa Pacific University, Azusa, CA, United States; ^2^ Noyes Laboratory of Chemical Physics, California Institute of Technology, Pasadena, CA, United States

**Keywords:** ATP, ATP synthase, ADP release, single-molecule tracking, single-molecule theory

## Abstract

A model-based method is used to extract a short-lived state in the rotation kinetics of the F_1_-ATPase of a bacterial species, Paracoccus denitrificans (PdF1). Imaged as a single molecule, PdF1 takes large 120^*ø*^ steps during it rotation. The apparent lack of further substeps in the trajectories not only renders the rotation of PdF1 unlike that of other F-ATPases, but also hinders the establishment of its mechano-chemical kinetic scheme. We addressed these challenges using the angular velocity extracted from the single-molecule trajectories and compare it with its theoretically calculated counterpart. The theory-experiment comparison indicate the presence of a 20*μ*s lifetime state, 40^*o*^ after ATP binding. We identify a kinetic cycle in which this state is a three-nucleotide occupancy state prior to ADP release from another site. A similar state was also reported in our earlier study of the Thermophilic *bacillus* F_1_-ATPase (lifetime ∼10μ s), suggesting thereby a common mechanism for removing a nucleotide release bottleneck in the rotary mechanism.

## 1 Introduction

In single-molecule imaging experiments, using small optical probes, steps are detectable in the rotation of single F_1_-ATPase motors. (Yasuda et al., 2001; Ueno et al., 2010). Complementing optical scattering techniques a variety of single-molecule imaging and manipulation techniques like optical tweezers, (Bustamante et al., 2021), atomic force microscopy (Uchihashi et al., 2011) or magnetic tweezers (Adachi et al., 2007) have been deployed in recent years to investigate the nanomechanical parameters of the bio-molecular motors. It was found that the steps are produced by a well-defined sequence of nucleotide binding, catalysis and release events in the three catalytic sites. The F_1_-ATPase rotary motor of the Paracoccus denitrificans species (PdF_1_) displays unusual behavior compared to the F_1_-ATPase in other species. During the rotation of PdF_1_, pauses (i.e., dwells) 120° apart are observed (Zarco-Zavala et al., 2020) which, due to the three active subunits in its *α*_3_ *β*_3_ ring, correspond to a single step per subunit in the 120° rotation cycle. In contrast, F_1_-ATPase in *E. Coli* bacteria, yeast and mammalian mitochondria were all seen to produce further smaller steps. (Bilyard et al., 2012; Steel et al., 2015; Kobayashi et al., 2020). PdF_1_ eluded previous efforts to resolve smaller rotation steps even when probed in a wide range of ATP concentrations. The interpretation of the three-step rotation is that the ATP binding dwell, the catalytic dwell and product release dwell in the three active subunits coincide up to a shift of ±120°, even at micromolar ATP concentrations. (Zarco-Zavala et al., 2020). The observations yielded constraints that prompted Noji and coworkers to suggest several possible rotational kinetic schemes. So, the detection of further smaller steps is desirable for its potential to help distinguish among rotary kinetics of PdF_1_, as was demonstrated previously in a series of single-molecule experiments by Noji and coworkers on the Thermophilic *bacillus* PS3 F_1_-ATPase (ThF_1_). (Adachi et al., 2007; Watanabe et al., 2012).

**FIGURE 1:**
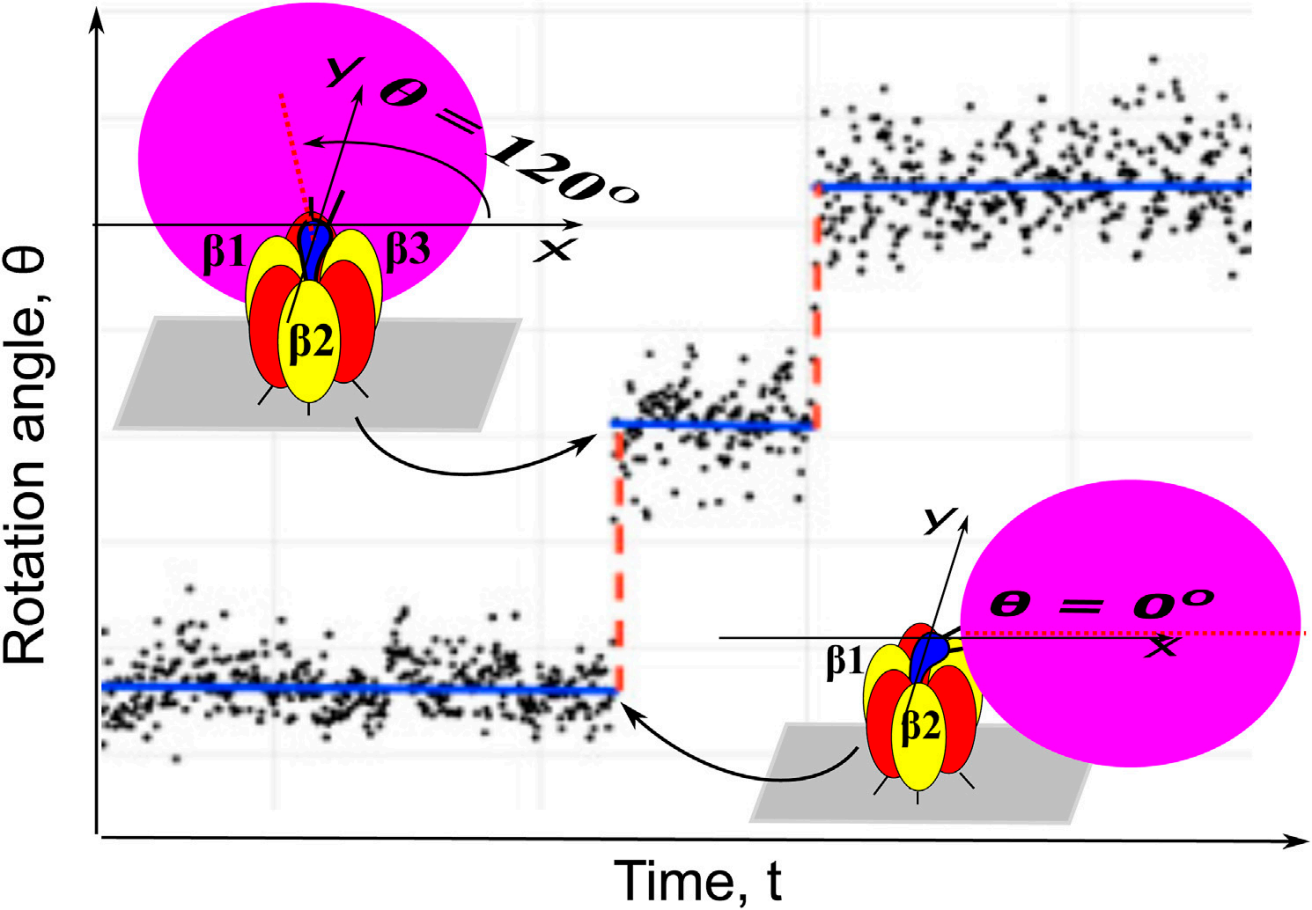
Dwells (blue solid base lines) and steps (red dashed lines) of a single-molecule trajectory of PdF_1_ imaged by attaching a nanocrystal probe (“bead”) to the rotor shaft of the motor. The probe undergoes Brownian fluctuations, hence the angles in the single-molecule image (black dots) deviate from the base lines but the large 120° stepping of the motor is still clearly seen in the imaging trajectories.

Recently the ThF_1_ rotary motor mechanism was elucidated using a theory-guided analysis (Volkán-Kacsó et al., 2019) achieving an increase in the effective time-resolution of stable rotation trajectories. We consider now similar high-quality trajectories for PdF_1_, seen in Figure 1, recorded via a single-molecule imaging probe attached to the *γ* rotor shaft of the nanomotor. We analyze brief stepping regions between dwells during which the propagation of coupled domain motions in the *α*_3_ *β*_3_ ring and the *γ* shaft produce short time events that is potentially “picked up” by the small optical probe (“bead”). These events tend to remain undetected due to time resolution limits of the apparatus and the “blurring” by the Brownian fluctuations of the probe. Angular histograms are “blind” to such short events and so instead, a model-based data analysis method using velocity-like statistics from stable trajectories with a “population” size of about 1,000 steps can reliably detect them. This approach was recently demonstrated (Volkán-Kacsó et al., 2019; Le and Volkan-Kacso, 2021) using the discretized angular velocity profile in ThF_1_ at high ATP concentration which led to the detection of a short-lived state in the step regions following ATP binding to the motor. The latter was postulated to be a three nucleotide occupancy state with lifetime corresponding to that of the fast ADP release, noting that Walker and co-workers also resolved a three-occupancy structure in F_1_-ATPase. (Menz et al., 2001).

In Figure 1 the substantial fluctuations of the angular position of the bead image are seen being superimposed on dwells and 120° steps in the rotation of the PdF_1_. Like in a previous work (Volkán-Kacsó et al., 2019) one main purpose in the paper is to extract information on a hidden state lifetime and on its most probable angular position, and give a procedure for extracting that information from the single-molecule imaging data with large fluctuations of the optical probe and of the protein. The method involves the solution of a set of relevant “Markov-Fokker-Planck” equatios with reaction terms and the Brownian fluctuations. It is a special case of a general framework with multiple states originally proposed by Oster and coworkers (Xing et al., 2005).

In the present article we describe an enhanced version of the angular velocity technique proposed in an earlier study (Volkán-Kacsó et al., 2019) which we apply in individually imaged PdF_1_ motors to achieve increased time resolution. Previously, angular velocities in F_1_-ATPase rotation were extracted by Frasch and co-workers from single-molecule observations using nanorod probes in a pioneering work. (Spetzler et al., 2009). They analyzed the mean rotational velocity during step regions between subsequent dwells and found it to be angle-dependent (Sielaff et al., 2016), and this angle dependence is exploited in the velocity technique to detect a hidden state.

In contrast to the previously described technique (Volkán-Kacsó et al., 2019; Le and Volkan-Kacso, 2021) the current enhanced method employed in this work is designed to be used when the time step of the imaging apparatus is significantly larger than the lifetime of a “hidden” event in the protein, as in the case of the 100 *μ*s time step used during PdF_1_ imaging. A further novelty of this method is that it is applicable when the time step is much larger than the presumably microsecond relaxation times of the large-scale motions in the motor (cf. ref. (Haran and Mazal, 2020). and references listed therin). A longer imaging time in PdF_1_ analyzed in the current work is needed due to the limited tolerance of the biomotor to strong incident light (compared to a 10 *μ*s imaging time step for the very robust ThF_1_ used in our previous work (Volkán-Kacsó et al., 2019)) and the small cross-section of the 40 nm optical scattering probe. We note that several previous works elucidated the release of P_i_ in the chemo-mechanical coupling scheme of the F_1_-ATPase (Okazaki and Hummer, 2013; Li et al., 2015; Ricardo et al., 2020), but the current work focuses on the release of the other product, ADP. Arguably, the ADP release latter presents challenges to both atomistic simulations and experiment: it is a slow process on the timescale of current simulations and it is study is hampered the necessary of purging hydrolyzed ADP in single-molecule experiments to prevent the ADP inhibited state.

## 2 Theory

### 2.1 Two-state model for step regions between dwells in PdF_1_ rotation

**FIGURE 2:**
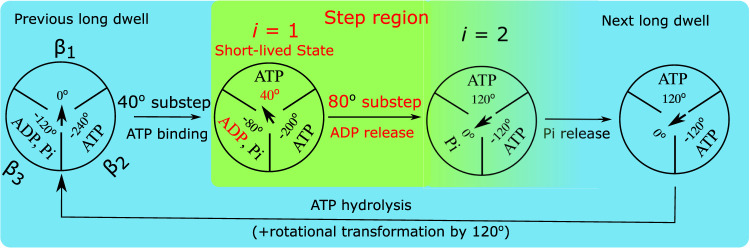
Diagram of the proposed chemo-mechanical rotation kinetic scheme for the PdF_1_ at micro-to milimolar ATP concentration. The circles represent a view of the PdF_1_ from the membrane side and the arrows in the center represent the angular position of the *γ* shaft relative to the *α*_3_ *β*_3_ ring. In the step region between two subsequent dwells there are two states: a “hidden” short-lived three-nucleotide state (*i* =1) and the final state (*i* =2) with its *β*_2_ subunit containing a Pi (Zarco-Zavala et al., 2020) an ADP. Subsequently, at the end of the next dwell the system proceeds to the following step region triggered by the binding of ATP to subunit *β*_2_. The rate constant *k*_*f*1_ stands for ADP release, which occurs before Pi release.

The proposed kinetic scheme in Figure 2 and the associated theoretical model of the F_1_-ATPase rotation described in this section are valid from physiological millimolar to the micromolar ATP concentrations, though not at picomolar concentrations (Adachi et al., 2012). We note that in Figure 2 the nucleotide occupancy varies between two and three. For rotation at micromolar ATP concentration the bottleneck process in the kinetic cycle is the binding of ATP, (Zarco-Zavala et al., 2020), so it produces long, well-resolved dwells in the single-molecule rotation. In the current analysis we use the theoretical method to treat experimental rotation data at millimolar concentrations (25 *μM*), although it can be readily generalized to higher concentrations, as demonstrated elsewhere. (Volkán-Kacsó et al., 2019).

Accordingly, in the proposed kinetic scheme in Figure 2, the end of the dwell coincides with an ATP from the solution binding to the motor enzyme and our goal is to investigate the presence of a hidden state in the subsequent transitions. Previously, Noji et al. have suggested that such a state in the transition might exist. (Zarco-Zavala et al., 2020). In Figure 2 this hidden state (identified with index *i* = 1) presumably occurs shortly after ATP binding, but before the release of products, and so is a three nucleotide occupancy state. State *i* = 2 is the final state during a step region, it will be the state after ADP release in subunit *β*_3_. In an alternative kinetic scheme Noji and coworkers also suggested that Pi might be released before ADP, (Zarco-Zavala et al., 2020), a possibility we also consider in a later section.

The probe, ranging in size from tens of nm’s to *μ*m’s, is subject to drag from the water and from the surface to which the *α*_3_ *β*_3_ stator is bound (cf. Figure 1). The probe’s friction and the internal friction in the motor protein (de Sancho et al., 2014) during rotation is described by an effective diffusion constant *D* (it is values used for calculations are provided in the “Results” Section). In the current implementation of Oster’s model, in state *i* with dwell angle *θ*_*i*_, the probe is subject to elastic torque −*κ*_*r*_ (*θ* −*θ*_*i*_) by the motor’s *γ* rotor to which it is attached via linkers. At micro to millimolar ATP concentrations the system follows a unique cyclic sequential kinetic pathway (Watanabe et al., 2013; Volkán-Kacsó and Marcus, 2015), without any ‘branching’, as in the scheme in Figure 2. The stepping regions from one dwell to the next in the single-molecule trajectories is described by a two-state kinetics, in which state *i* = 1 corresponds to the postulated short-lived state from which the system jumps to a state *i* = 2 by releasing an ADP. The probability densities *ρ*_*i*_ (*θ*, *t*) of being in state *i* follow a diffusion-reaction equation (Oster and Wang, 2000; Volkán-Kacsó et al., 2019), in which the “source” and “sink” terms for state *i* are angle-dependent transfer rates, *k*_*f*,*i*_ (Volkán-Kacsó and Marcus, 2017), that appear at as the last terms in the equations.

$$\frac{\partial \rho_{1}}{\partial t}=D\frac{\partial }{\partial \theta }\left[\frac{\kappa_{r}}{k_{B}T}\left(\theta -\theta_{1}\right)\rho_{1}\right]+D\frac{\partial^{2}\rho_{1}}{\partial \theta^{2}}-k_{f,1}\left(\theta \right)\rho_{1}$$ (1)

$$\frac{\partial \rho_{2}}{\partial t}=D\frac{\partial }{\partial \theta }\left[\frac{\kappa_{r}}{k_{B}T}\left(\theta -\theta_{2}\right)\rho_{2}\right]+D\frac{\partial^{2}\rho_{2}}{\partial \theta^{2}}+k_{f,1}\left(\theta \right)\rho_{1}$$ (2)

The back rate constants are negligible since hydrolyzed ADP is purged from the solution to prevent the ADP inhibited state in signle-molecule rotation imaging. In these equations, in the first two diffusive terms on the right hand side, at temperature *T* (*k*_*B*_ is the Boltzmann constant), one allows for deviations (fluctuations) of the monitored angle *θ* from dwell angles *θ*_1_ or *θ*_2_, as the former is subject to a parabolic potentials centered at the dwell angles. In a given state *i*, the angular position *θ* in Eqs. 1, 2 is described by a and Ornstein-Uhlenbeck process (van Kampen, 1981) with an equilibrium displaced from the origin by *θ*_*i*_. In Figure 2 state *i* = 2 is described as having a dwell angle of *θ*_2_ = 120^o^.

When the system jumps from *i* = 1 to *i* = 2, i.e., from one paraboloid to another one, it does so according to an angle-dependent rate constant *k*_*f*_(*θ*) with a functional form and parameters given in the Si and the ‘Results’ Section. The duration of this transition is so short on the timescales of single-molecule experiments that the probe (bead) is virtually motionless during this transition. The rotor shaft and linker therefore will torsionally be distorted (elastic distortion) and the system then slowly relaxes as the bead now “catches up” to the next dwell. Noji and co-workers had previously described a similar picture of an elastically coupled probe in the ThF_1_ using Langevin-type equations (Watanabe et al., 2013) based on an earlier work by Sumi and Marcus (Sumi and Marcus, 1985).

In the PdF_1_ rotation during the 120° jumps from one long dwell to the next in Figure 2, the step region is initiated by the completion of the ATP binding transition from the outside to the interior binding pocket. Recalling that this transition is much faster than the single-molecule imaging time step the system is initially found in the ATP-bound state *i* = 1, a 3 occupancy state. The previous dwell was presumably long compared to the optical probe’s characteristic relaxation time $\tau =k_{B}T{\left(\kappa_{r}D\right)}^{-1}$ . (Volkán-Kacsó et al., 2019). which means that the probe completely equilibrated, and so the proper initial condition for solving Eqs. 1, 2 is $\rho_{1}\left(\theta ,0\right)=A_{1}\exp\left[-\left(\kappa_{r}\theta^{2}\right)/\left(2k_{B}T\right)\right]$ , normalized as $A_{1}=1/\sqrt{2\pi k_{B}T/\kappa_{r}}$ and *ρ*_2_ (*θ*, 0) = 0. The slow bead is still in the previous long dwell, even though the motor has already transitioned to the 3-occupancy state: the probe has not yet responded to this sudden change. Prior to this step region, the system waited for the ATP from the solution to collide with the PdF_1_, and so the dwell is termed ATP waiting dwell. It is the rate-determining process at the micromolar ATP concentration used in the PdF_1_ single-molecule experiments.

The system next proceeds to jump to state 2, upon the fast release of ADP from subunit *β*_3_, a process described by rate constant *k*_*f*,1_(*θ*). At this point, even though the stable dwell angle for state 2 is *θ*_2_ = 120° the angle of the probe is still in the step region, i.e., 0 < *θ* < 120°, and subsequently it tends to the next dwell at 120°. In doing so the imaging probe responds with a characteristic relaxation time of *τ*.

In state 2 only the Pi is left in the pocket (Figure 2). The Pi release is relatively slow (millisecond range), so it will occur only after the system has relaxed to the next long dwell. In that dwell than the ATP hydrolysis (in another pocket to which ATP bound 240^*o*^ ago) will also occur, followed by ATP binding to the empty pocket, completing the cycle.

### 2.2 Theory of angular velocities in step regions

The key entity in the present procedure is the discretized angular velocity which is extracted from single-molecule tracking experiments. (Spetzler et al., 2009). In the experiments considered in the current analysis the imaging apparatus records successive images with a time step Δ*t* (the time to capture a video “frame”) from a scattering nano-probe attached to the rotor shaft. (Noji et al., 1997). The angular position of the probe is estimated for each frame (Yasuda et al., 2001) and yields a single-molecule trajectory as a series of time-dependent angular positions {*θ*(*t*)} for a set of discrete times {*t*_0_, *t*_0_ + Δ*t*, *t*_0_ + 2Δ*t*, … }. When divided by the time step Δ*t*, an angular jump Δ*θ* = *θ* (*t* + Δ*t*) − *θ*(*t*) from an angular position at time *t* to an angular position at time *t* + Δ*t* yields a “instantaneous” discretized velocity at time *t* + Δ*t*/2, *v* = (Δ*θ*/Δ*t*). We note that the center point of the subsequent frames is used in this definition. In this work it will be simply called angular “velocity.”

In the theory, solving Eqs. 1, 2 yields the *ρ*_*i*_ (*θ*, *t*) which are then used to calculate an angular velocity probability distribution function *ρ*(*v*|*θ*) of the system having a discretized velocity *v* at a given angle *θ*. For the *ρ*(*v*|*θ*) distribution, an averaging over the mean time *T*_*S*_ of a step region is completed, noting that the value of *T*_*S*_ is extracted from experiment by averaging over the times the system spends completing the step regions. At the end of a step region the mean *θ* angle tends asymptotically to the next long dwell (cf. Supplementary Figure S10 or Supplementary Figure S14), in the analysis of experimental trajectories we consider a transition complete once the average angle is within about 5^o^ of the new dwell. Then the experimentally determined *T* time is used in the theoretical calculations, for consistency between the data analysis and theory.

In the theory the averaging over *T*_*S*_ yields the relative contributions *p*_*i*_(*θ*) from states *i*, $p_{i}\left(\theta \right)=\int_{0}^{T_{S}}dt\rho_{i}\left(\theta ,t\right)/A\left(\theta \right)$ , where $A\left(\theta \right)=\sum_{i=1}^{2}\int_{0}^{T_{S}}dt\rho_{i}\left(\theta ,t\right)$ . Then, if we define the short-hand notation for velocities $v_{pi}\left(\theta \right)=-\left(\theta -\theta_{i}\right)/\Delta t\times \left(1-e^{-\Delta t/\tau }\right)$ , a summation over all states yields, (Volkán-Kacsó et al., 2019), as described in the SI, a sum of Gaussian with peaks (and so means) at velocities *v*_*pi*_,

$$\rho \left(v|\theta \right)=\overset{2}{\underset{i=1}{\sum }}\;\frac{p_{i}\left(\theta \right)}{2\sqrt{\pi D/\Delta t}}\exp\left\{-\frac{{\left[v-v_{pi}\left(\theta \right)\right]}^{2}}{4D/\Delta t\left(1-e^{-\tau /\Delta t}\right)}\right\}.$$ (3)

Using *ρ*(*v*|*θ*) to average over all states *i* = 1 and *i* = 2 yields the mean angle dependent angular velocity, $\left\langle v\right\rangle \left(\theta \right)=\int_{-\infty }^{\infty }v\rho \left(v|\theta \right)dv$ , which results in the expression,

$$\left\langle v\right\rangle \left(\theta \right)=\frac{1}{\Delta t}\left(1-e^{-\Delta t/\tau }\right)\overset{2}{\underset{i=1}{\sum }}\left(\theta_{i}-\theta \right)p_{i}\left(\theta \right).$$ (4)

Considering only one term in the sum from Eq. 4, i.e., when the system is found in a dwell corresponding to a particular biochemical state *i* (for that state *p*_*i*_ = 1) an analytical solution is found for the mean velocity, i.e., $\left\langle v\right\rangle \left(\theta \right)=v_{pi}\left(\theta \right)$ . The theoretical model predicts that: 1) the velocity is a linear function of the rotor angle, 2) the slope of this line is given by $\left(-1/\Delta t\right)\left(1-e^{-\Delta t/\tau }\right)$ , and 3) the intercept of the line yields the dwell angle *θ*_*i*_. These features are verified using single-molecule experiments in the Results and Discussion section.

## 3 Results

### 3.1 Application to single-molecule data: angular velocity distributions reveal hidden state

The procedure used to detect the short-lived hidden state in the single-molecule trajectories involves an effective fitting of the theory to the experimental data. The basis of the fitting procedure is the angle dependent angular velocity which is both calculated in the theory as described in the previous section and extracted from single-molecule data. In the current section a procedure is described for the theory-experiment comparison is used in order to demonstrate the presence of the short-lived state in the step regions in single-molecule trajectories.

**FIGURE 3:**
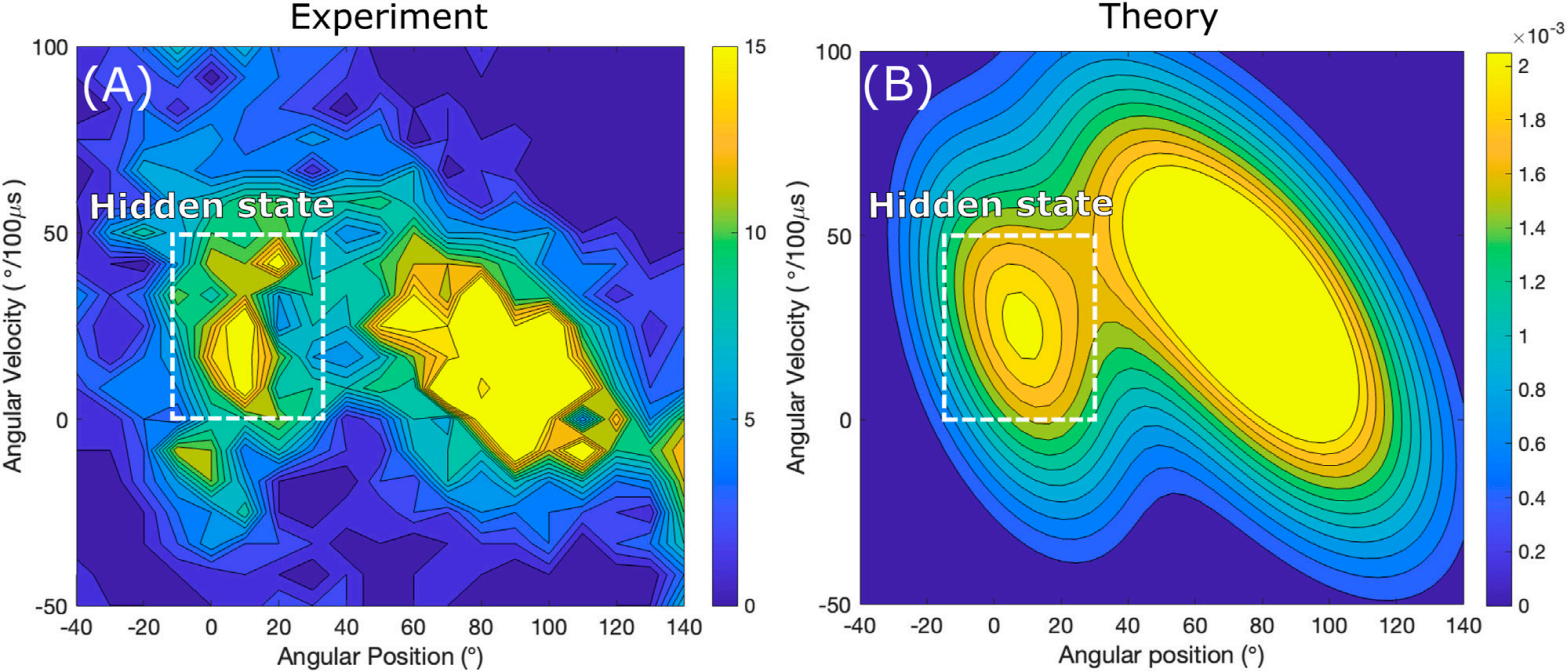
Contour plots of histograms of rotation in the step regions as a function of angular position *θ* (horizontal axis) and angular velocity *v* (vertical axis). Binned data from trajectory **(A)** is compared with probability distribution calculated using a two-state theory **(B)**. The smaller “island” feature showing an increased density of events is due to the short-lived state in the step region. The experimental data is courtesy of **H**. Noji and coworkers (Zarco-Zavala et al., 2020).

In particular, using data in a trajectory, one can build the experimental angular velocity-angle histogram *N* (*v*_*j*′_|*θ*_*j*_) plotted in Figure 3A. The procedure, described in detail in Supplementary Section SV, involves grouping the velocity data {*v*(*t*)} from multiple step region events in angular bins centered at discretized angles *θ*_*j*_ and angular velocity bins centered at *v*_*j*’_. For comparison, the corresponding theoretical angular velocity probability distribution function at a given *θ* angle in the step region *ρ*(*v*|*θ*) is plotted in Figure 3B.

The angular velocity-angle distribution in the step region extracted from a trajectory in Figure 3A exhibits two areas of increased probability density profiles. It shows the evidence of a short-lived state in the step region. In a two-state model in Figure 3B each of these areas with the appearance of “mountain hills” corresponds to a high probability that the system is in a particular state: the smaller area early in the step region corresponds to the short-lived state and the larger area closer to the end of the step region corresponds to the state in the next dwell, states *i* = 1 and *i* = 2 on Figure 2, respectively. Conversely, a one-state model, which would assumes no short-lived state in the step region, would only produce a single hill, and so the smaller island would not be present in Figure 2.

### 3.2 Hidden state: a 30 *μs* substep 40° into the step region

In the previous section the angular velocity-angle probability distribution was used to provide a qualitative evidence for the presence of a short-lived state detected as an increased density in Figure 3A. The goal of this section is a quantitative analysis of the short-lived state, in order to extract its specific life time and angular position in the step region. To do so the mean angular velocity angular profile is both computed in the theory and calculated using experimental data, and the two profiles are then fitted. Calculating the experimental average velocity entails an averaging over the angular velocity on Figure 3A which for single-molecule trajectories has the benefit of reducing statistical ‘fluctuations’ which are present due to finite data counts in the step region.

Accordingly, in the analysis of the experimental trajectories, the mean of the angular velocity ⟨*v*⟩(*θ*) is not assumed to be constant, instead it is angle dependent. In the single-molecule trajectories, this mean angular velocity at an angle *θ* is calculated by averaging over many rotation cycles. (Volkán-Kacsó et al., 2019). The averaging follows the procedure described by Frasch and co-workers (Spetzler et al., 2009), in which angular velocities for angles centered at bin angle *θ*_*i*_ are calculated from multiple stepping events.

**FIGURE 4:**
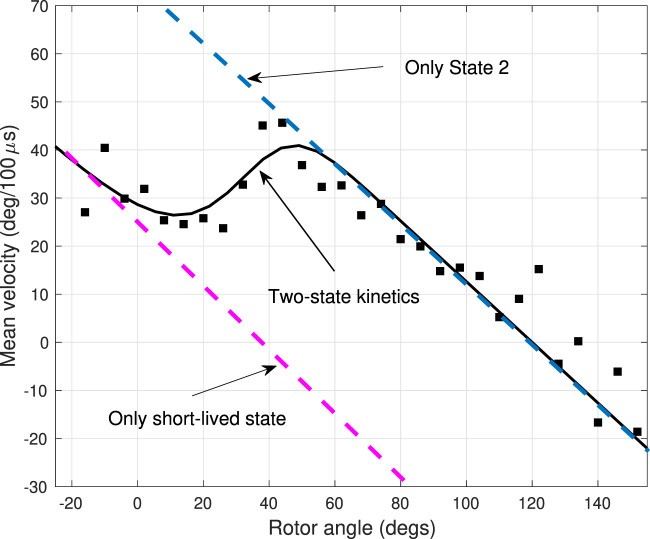
Experimental and theoretical average angular velocity profile for a trajectory also used in Figure 3. Black squares indicate the average angular velocity in the step regions between dwells, extracted from the single-molecule experiments. The theoretical velocities assuming a two-state system with a short-lived intermediate state and a longer-live final state are shown as black solid lines. Dashed lines show theoretical calculations in the presence of only one of the states.

In the theory, the equivalent average angular velocity as a function of rotor angle $\left\langle v\right\rangle \left(\theta \right)$ is evaluated from *ρ*(*v*|*θ*) for each component *i*, as described in a previous section. The theoretical and experimental angular velocity curve were overlaid on the experimental counterpart in Figure 4. The model parameters used in the calculations are listed in Table 1. The angle-dependence of the forward rate constant *k*_*f*1_(*θ*) is assumed to be exponential over a range of angles, according to the elastic molecular transfer mechanism (Volkán-Kacsó and Marcus, 2015), as well as earlier single-molecule stalling (Watanabe et al., 2010) and controlled rotation (Adachi et al., 2012) experiments. The specific angle-dependence of *k*_*fi*_ and *k*_*bi*_ are also described in the SI. The theoretical mean angular velocity was calculated using spring constants *κ*_*r*_ and diffusion coefficients extracted from trajectory dwells as described in the SI. Since the ADP concentration is kept low in the solution, ADP binding is slow, so in Eqs. 1, 2 we assume that the back rate is negligible, *k*_*b*1_(*θ*) ≈ 0.

**TABLE 1:** Physical quantities in the 2-state model used for three different PdF_1_ specimens at 25 *μM* ATP concentration. Trajectory A yielded the results from Figures 3, 4.

Trajectory	*κ*_*r*_ (pN⋅nm)^*a*^	D (deg^2^/*μs*)^*a*^	*θ*_1_ (°)^*b*^	1/*k*_*f*1,0_ (*μs*)^*b*^
A	26	4.9	40	25
B	37	4.1	38	27
C	45	3.1	45	20

In the analysis, then there are only two “fitting parameters”, the dwell angle of the hidden state *θ*_1_ and its ‘life time’ 1/*k*_*f*1,0_. The method of fitting the theoretical curves to experimental velocities involved the calculation of the $\left\langle v\right\rangle \left(\theta \right)$ curve on a discrete ‘grid’ of possible pairs of parameters (*θ*_*i*_, *k*_*f*0_). On this grid then a search was performed for the global maximum of a goodness-of-fit scoring function, the inverse of mean square deviation between calculated and experimental velocities. Using this procedure, a lifetime of 1/*f*_*f*0,1_ ≈ 25*μs* and dwell angle of *θ*_1_ ≈ 40° were extracted. In addition to velocities from the trajectory from Figure 3 (trajectory “A”), two additional trajectories (“B” and “C”) were also analyzed and yielded similar results. In Table 1 the *θ*_1_ and 1/*k*_*f*1,0_ values are listed for the three different trajectories A-C from the mean angular velocity based method of fitting theory to experiment (cf. Figure 4).

^*a*^ Quantities extracted from the dwells. ^*b*^ Quantities for the rate constant of the hidden state (cf. Eqs. 1, 2) extracted from the step regions using the 2-state model.

In the angular velocity profile, there is a marked qualitative difference between a one-state and a two-state system. In the former, a single equation *∂ρ*_2_/*∂t* = *Dβκ*_*r*_ (*∂*/*∂θ*)(*θ* −*θ*_2_)*ρ*_2_ + *D∂* ^2^ *ρ*_2_/*∂θ* ^2^ yields a linear angular velocity vs angle profile, as predicted by the theory of velocities shown in Figure 4 (blue dashed lines). In the latter, the presence of the additional state in the step region produces a dip in the velocity profile, where otherwise would be continuously decaying linear function. In the model the slowdown is due to the short-lived state leading to the momentary ‘dwell’ of about 25*μs*. The dip in the angular velocity, a qualitative hallmark of the short-lived state, is consistently present in all three trajectories in Figure 4. Indeed, the effect is so strong that in an angular range from about 30^o^ to 60^o^ the slope of the angular velocity vs angle curve is reversed (made positive) by the presence of the short-lived state. Quantitatively, the states 1 and 2, i.e., the short-lived and the final states, appear to be quite distinct in the angular velocity profile: the linear angular velocity-angle relations corresponding to the two states (dashed lines on Figure 4) have a significant shift in their intercepts, and this shift corresponds to the difference between the dwell angles of the two states.

## 4 Discussion

### 4.1 Features in the angular velocity vs angle and their relation to the short-lived state in the step region

According to the two-state model for PdF_1_, in Figure 3 the larger of the two probability density features are due to state 2, the catalytic state according to Figure 2, which is associated with the first part of the dwell after the step region. A smaller “hill” is due to the system being in state *i* = 1 which is the clear signature of the “hidden” state in the step region.

When the F_1_-ATPase fluctuates in a stable dwell state, its average angular velocity is proportional with the angular displacement from the dwell angle. Due to the elastic nature of the coupling between the probe and the motor, the torque behaves as a restoring force: it is proportional with the angular displacement. Then, since the system is in the overdamped (Brownian) regime, the average angular velocity is also proportional with the angular displacement. The proportionality is best illustrated by plots generated from data in the dwells along trajectories shown as red dots in Supplementary Figure S16. The negative slope of the angular velocity-angle line in a state is determined by the elastic constant and viscous drag of the probe, according to Eq. 4. The intercept of the velocity-angle line is identical with the dwell angle of the current biochemical state of the motor, as is verified in the mean angular velocity *versus* angle plots in the dwells at *θ* = 0° in Figure 4. On Figure 4 the first data points in the step region are associated with the short-lived state *i* = 1. They give rise to a line on the average velocity-angle plot with an intercept of *τ*_1_ ≈ 40°.

When the system transitions from one state to another, the mean angular velocity is a weighted average of the mean velocities corresponding to the two states (cf. Eq. 4). In a range of rotor angles between the two dwells *θ*_1_ and *θ*_2_ the system can be found in either state 1 or state 2, so the probabilities of being in either of these states is comparable. Each state will produce it is own angular velocity-angle line with the same slope, but different intercepts: *v*_*p*1_(*θ*) is negative and *v*_*p*2_(*θ*) is positive and these components give rise to a bimodal angular velocity distribution. So, as *θ* increases, the contribution from the second (positive) peak increases and so the overall mean angular velocity $\left\langle v\right\rangle \left(\theta \right)$ will also increase. It yields the right “wall” of the well in Figure 4 in the range between 20° and 50°.

Finally, starting at about 50° the system settles into state *i* = 2, resulting in the ‘final descent’ of the mean angular velocity. The 2-state interpretation of the angular velocity in Figure 4 implies that the slopes of these decaying lines are the same as the slope of the average velocities in the dwell at *θ*_1_ = 120° after the step region. In other words, the data points that give rise to the decaying lines are interpreted as the system being in the ‘final’ state *i* = 2, which is the same as the state associated with the dwell after the step region. While the system switches quickly to the final state, the probe is slower to respond, so giving rise to the decaying $\left\langle v\right\rangle \left(\theta \right)$ line.

### 4.2 Chemo-mechanical scheme of PdF_1_F_0_-ATPase in relation to other members of the F_1_-ATPase family

The detection of the short-lived state that occurs after ATP binding elucidates the mechano-chemical kinetic scheme of PdF_1_. It adds another detectable step to the dwell in each 120° cycle represented in Figure 2, resulting in a net total of 6 steps in a full rotation cycle. According to this scheme, there are two events that produce mechanical stepping: a ∼40° substep by ATP binding and ∼80° substep by ADP release. The other two events, ATP hydrolysis and Pi release appear to occur during the dwells and so they do not produce obviously detectable substeps in the single-molecule imaging experiments.

The ADP release first scenario (Zarco-Zavala et al., 2020) is supported by the similarity between the short-lived state in PdF_1_ and the three occupancy state in ThF_1_ (Volkán-Kacsó et al., 2019), noting that Walker and co-workers also resolved a three-occupancy structure in F_1_-ATPase. (Menz et al., 2001). In both PdF1 and ThF1 the velocity-based approach yielded an increased temporal resolution, which in turn was used to detect a hidden state that appears to be in both cases associated with the ADP release after ATP binding occurred in another subunit. The similarity of the hidden state in the PdF_1_ and ThF_1_ is apparent: in both enzymes, the state appears roughly 40° following the ATP binding dwell and the life times extracted are also similar, about 10 *μ*s for ThF_1_ and 20 *μ*s for PdF_1_. Moreover, our analysis shows that the exponential angle dependence of the rate constant of the ADP release process, when assumed to have the same exponential coefficient as the slow and fast ADP release in ThF_1_, yield a good fit for the average angular rate vs angle function. Conversely, an angle-independent rate constant is not consistent with experiment (cf. Supplementary Figure S15). So, an alternative order of events (Zarco-Zavala et al., 2020), where Pi is released before ADP, is not supported by our findings.

### 4.3 Allosteric ADP release: a possible common mechanism and its role in the function of F_1_-ATPase

The short-lived state following ATP binding appears to be present in both ThF_1_ and PdF_1_. This accelerated ADP release then, driven by an allosteric mechanism of ATP binding to another site, is found to be present in both ThF_1_ and PdF_1_. A possible implication is that the concerted mechanism that accelerates ADP release had a common origin in the F-ATPases. Further studies of various F-ATPases and other rotary motors may reveal the presence of the allosteric acceleration of product nucleotide (e.g., ADP) release.

An additional substep in each 120^o^ of rotation “breaks” the large 120^o^ step in both PdF_1_ into two smaller substeps. Similarly, the 80^o^ step in ThF_1_ is broken into two 40^o^ substeps. So, both motors take more numerous and smaller substeps as previously observed, so during their rotation these motors need not undergo extensive twisting stresses as they switch from one biochemical state to the next. It was suggested that the build-up of torsional elastic tension resulting in temporary stored elastic energy (Oster and Wang, 2000) occurs in both single-molecule imaging experiments on the F_1_-ATPase and the actual function of the full ATP Synthase. (Sobti et al., 2020). With a smaller energy penalty during each stepping, the F_1_F_0_-ATPase was shown to achieve a smoother and faster operation (Panke et al., 2001).

## 5 Conclusion

Using a model-based approach for the analysis of single-molecule rotation data in the step regions between dwells, a short-lived state was discovered in the rotation of the PdF_1_ motor enzyme. This state, previously undetectable due to its microsecond lifetime was shown to be a three nucleotide occupancy state, prior to ADP release. We note the advantages in microsecond temporal resolution presented in this work when compared with other classical binding techniques like surface plasmon resonance (SPR) (Crona et al., 2010) or AFM (Pérez-Domínguez et al., 2022) which are capable to measure dissociation constants of ATP or NADP + nucleotides, respectively, much above 10 microseconds (typically milliseconds). We found that the ADP release in F_1_-ATPase is triggered by the binding of ATP at another site. This allosteric mechanism of product nucleotide release by the binding of another nucleotide might be a common mechanism that the F-ATPase motors have used over the eons of evolution. In a “clever” way, likely favored by evolutionary mechanisms, the substrate nucleotide (ATP for hydrolysis) also acts as a catalyst for product release acceleration, thereby solving the problem of the high product affinity. This mechanism requires at least 3 active sites, a third site being necessary to accommodate the hydrolysis step prior to the allosterically catalyzed product release in the other two sites. In this sense, the F_1_-ATPase with its 3 active subunit is a minimal system for allosteric self-catalysis. By inactivating the other three sites in the F_1_-ATPase ring and using the proper number of subunits in the C-ring for efficient and robust energy conversion, the function of this self-catalyzed system was perfected.

## Data Availability

The data analyzed in this study is subject to the following licenses/restrictions: Data analyzed are a curtesy of the H. Noji lab (U. Tokyo). Requests to access these datasets should be directed to Sandor Volkan-Kacso, svk@caltech.edu.
