# Supplementary material for: Angle-dependent rotation velocity consistent with ADP release in bacterial F 1 -ATPase
Source: Front Mol Biosci. 2023 Aug 2;10:1184249. doi: 10.3389/fmolb.2023.1184249 (PMC10433373; doi:10.3389/fmolb.2023.1184249)
Supplement: Supplementary file 1 [file DataSheet1.pdf]

Supplementary Information for ”Angle-dependent rotation velocity consistent with ADP release in bacterial F_1-ATPase”

Nathan Suiter[†,‡,¶] and S. Volkán-Kacsó[*,‡,†]

†*Department of Mathematics, Physics and Statistics, Azusa Pacific University, Azusa, CA 91702, USA*

‡*Noyes Laboratory of Chemical Physics, California Institute of Technology, Pasadena, CA 91125, USA*

¶*Current address: Department of Statistics, University of Oxford, Oxford, UK*

E-mail: svk@caltech.edu

Rotational symmetry and correction

Prior to analysis, the recorded position of probe's centroid was examined to ensure that all samples behaved sufficiently similarly to each other to enable comparison, with rotational correction being performed as necessary. Fig. S1 shows the recorded positions of singular rotating PdF1, imaged using a gold quantum dot probe according to a method devised by Noji and coworkers.[1] Initially, these measurements indicated the existence of asymmetries between subunits within some samples. The existing irregularities were believed to be caused by nanoscale imperfections on the substrate surface rather than abnormal operation of the enzyme itself, hence, a rotational correction was performed by tilting the z-axis of the PdF1.

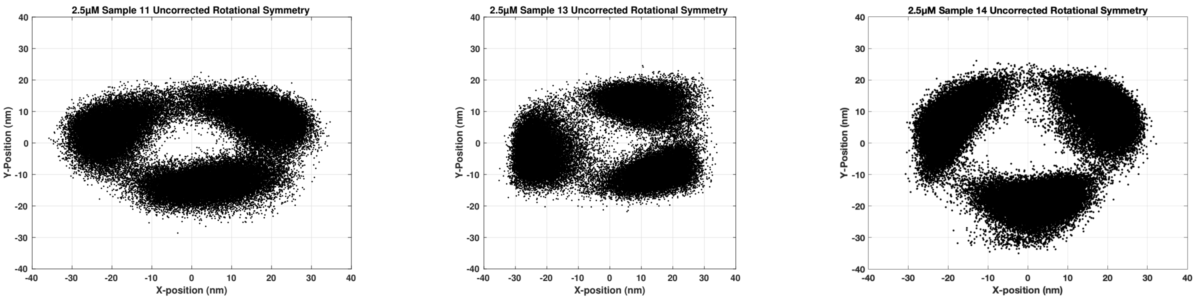

Fig. S1: Scatterplots of the uncorrected centroid position of the optical nano-probe attached to the rotor shaft of PdF1 in Cartesian coordinates for a) 2.5 μM Sample 11 b) 2.5 μM Sample 13 Scatter c) 2.5 μM Sample 14

Rotational correction was performed using an algorithm that optimized similarity between the three subunits, with an example output shown in Fig. S2. The goodness-of-fit peak corresponded to optimal tilting and rotation values relative to the experimental origin.

The correction contributed to comparability between subunits, as shown in Fig. S3. Despite some lingering differences after correction, subunits within respective samples yielded more consistent results overall, generally allowing for comparative analysis of subunits within samples.

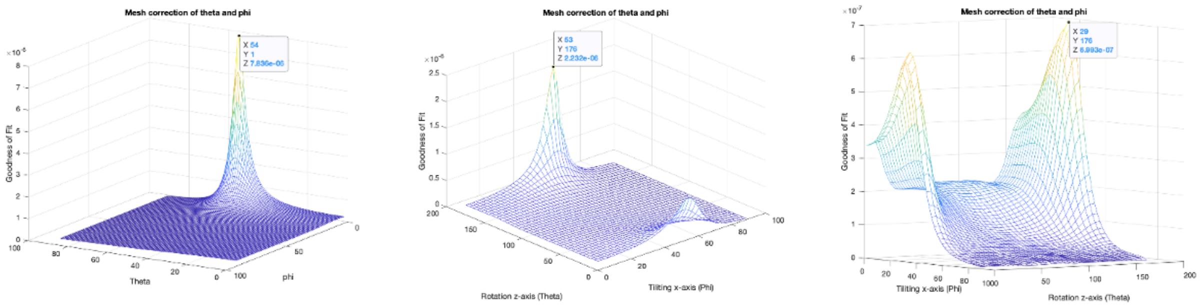

Fig. S2: 3D grid of generated goodness-of-fit theta and phi correction values for a) Sample 11 b) Sample 13 and c) Sample 14

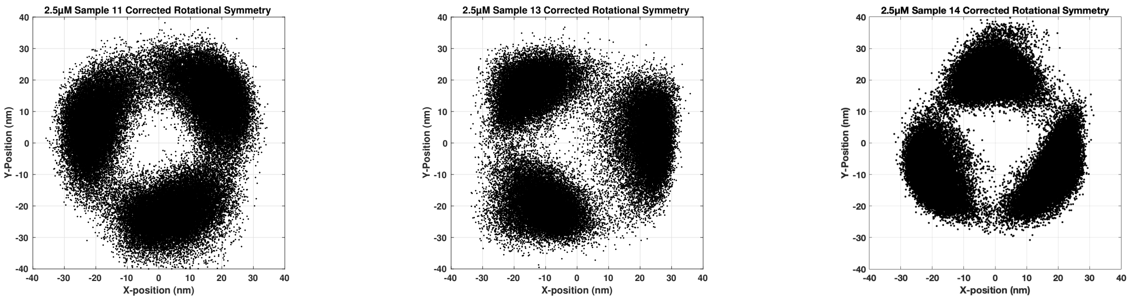

Fig. S3: Scatter plot of corrected centroid position for (a) Sample 11, (b) Sample 13 and (c) Sample 14

Torsional spring constant

Corrected data was used to calculate an improved estimate of respective systems' torsional spring constants. Original estimates shown in Fig. S4 are asymmetric with significant variances in subunit height and width; trends that do not make sense on account of being recorded in the same system. The data in Fig. S5 provides significantly more similarity between calculated torsional spring constants.

The calculated uncorrected and corrected spring constants can be seen in Table S1 and Table S2 respectively. While there is fluctuation in the calculated values for both uncorrected and corrected data, the corrected results have lower or equal variance for each sample. The subunits in Table S1 and S2 were sequenced according to the sequential magnitudes of spring constant as opposed to grouping by biological structure.

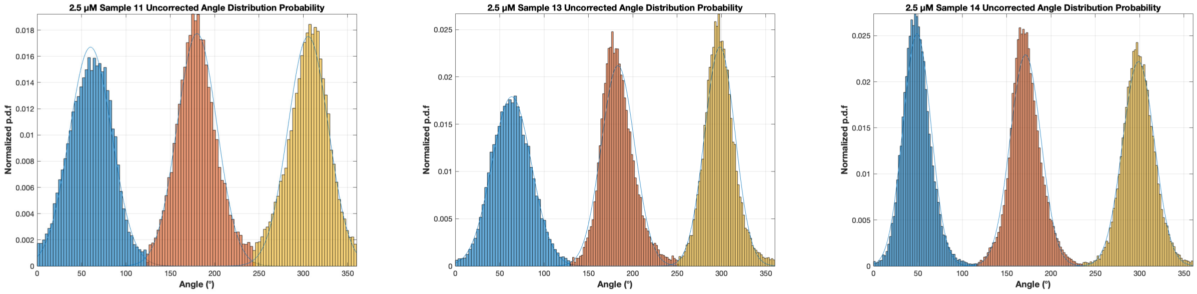

Fig. S4: Histogram of uncorrected angular position p.d.f. for subunits in a) Sample 11 b) Sample 13 and c) Sample 14

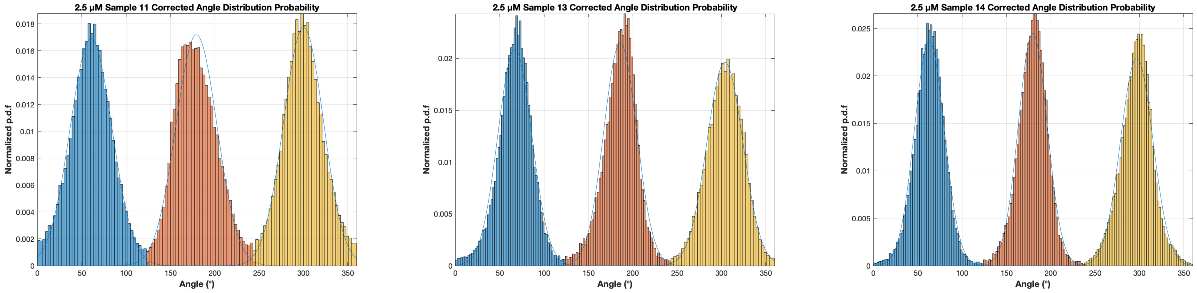

Fig. S5: Histogram of corrected angular position p.d.f. for subunits in a) Sample 11 b) Sample 13 and c) Sample 14

Table. S1: Uncorrected Torsional Spring Constant Values κ_r (pN·nm).

Trajectory	Subunit 1	Subunit 2	Subunit 3
A	24	26	27
B	27	38	45
C	42	44	54

Table. S2: Corrected Torsional Spring Constant Values κ_r (pN·nm).

Trajectory	Subunit 1	Subunit 2	Subunit 3
A	24	25	27
B	33	37	39
C	40	47	51

Angular fluctuations in the dwell

Examining dwell behavior shows that fluctuations are centered around chemical equilibrium at 0, as seen in Fig. S6, a result consistent with Brownian noise. Within Fig. S6, any perceived change in dwell magnitude is due to a smaller quantity of data points rather than an actual behavior. The distribution of angular position within dwells can be seen in Fig. S7, the samples mostly follow a Gaussian with a heavier right-tail and a recorded mean near zero.

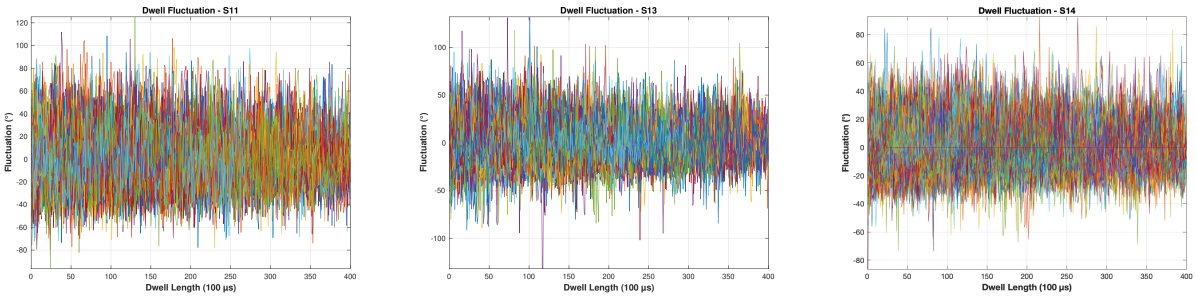

Fig. S6: Dwell fluctuation over time for a) Sample 11 b) Sample 13 and c) Sample 14

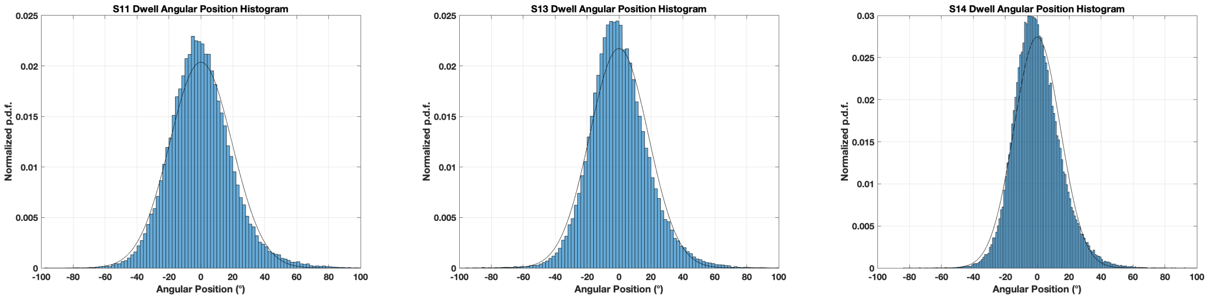

Fig. S7: Derived p.d.f. angular position distribution for a) Sample 11 b) Sample 13 and c) Sample 14

Method for separating dwells and transitions

An important part of evaluation is separating dwell and transition states. While determining state changes with absolute accuracy was not feasible, the method used to separate states

was primarily based on the energy equilibrium and likelihood theorems, with specific steps listed below.

1. Plotting cumulative angle vs time

Random fluctuations within the data prohibited direct examination of the probe's true behavior. Plotting the cumulative angle versus time proved better for manual separation of states as it provides a clear picture of local and global behavior at any given point. An example of a plot used within the procedure can be seen in Fig. S8.

2. Identifying definitive dwell states

Visually, dwell regions were majorly identifiable. In Fig. S8, the dwell areas are represented using a solid line. As dwells reflect when the enzyme is in chemical equilibrium, the net change in angle is zero. Practically there were some dwells that had a nonzero mean ($\pm$ 2°), but this was believed to be caused by insufficiently long dwells. While data at dwell edges proved to be challenging to separate, the general location of moderately long dwell states (around $n > 50$) was readily apparent.

Within regions that behave indistinguishably from normal dwell states, there are jumps far beyond the calculated variance that could be the enzyme undergoing a failed rotation or a measurement artefact. Comparatively, within transitions, there are occasionally, sequences where the probe spends an uncharacteristic amount of time in a transitions (1-2 ms), which is shorter than a normal dwell, but longer than most transitions. Within the scope of this paper, the former events were considered as dwells (as they did not have a significant effect the net dwell average), and events where the probe appeared to fluctuate about for greater than 1 ms were considered dwells, as the focus was with transitions which appeared to have an average 0.5 ms.

3. Evaluating the dwell variance

After identifying parts of the trajectory that were definitively in a dwell state, the next step was to identify the variation within dwells to determine the magnitude of fluctuation. The fluctuations can be extracted simply used the pdf as seen in Fig. S7.

4. Determining dwell state averages

Subsequently, individual dwells' energy equilibrium was obtained using the previously determined dwell points, verified by summing the normalised dwell points. Ultimately, the average appeared like the dashed line in Fig. S8, wherein fringe points fall above or below the line. For moderately long dwells are relatively long, the inclusion or exclusion of fringe points generally had a negligible effect, although it did provide an additional validation method in the form of ensuring a zero-sum output.

5. Identifying definitive transition states

As transitions are approximately 120°, visible as a dashed line in Fig. S8, the center points within transitions were generally apparent based on the dwell variance. Consequently, the center of a transition could be determined by using surrounding dwell averages and variances.

6. Deciding transition beginning criteria

Within trajectories the main difficulty arises in determining the beginning of transitions and end of dwells. Due to the variance, jumps at the end of dwells could be a part of a dwell or a new transition. As our focus was on transitions, the initial transition start point was considered to be the last data point that was above the dwell average without ever returning below it. During analysis focused solely on transitions, a lag time of 0.1-0.2ms was often used in an effort to minimize any dwell points in the fringe values.

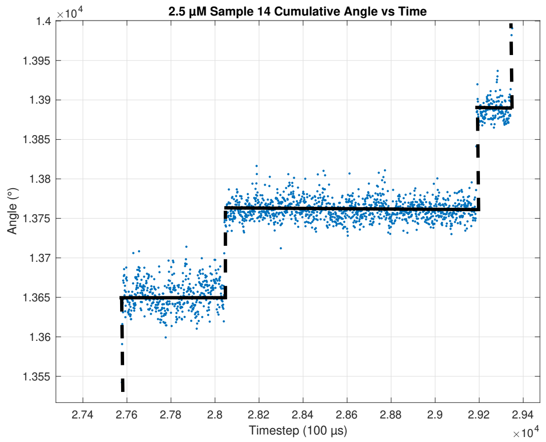

Fig. S8: Sample 14 Cumulative Angle versus Time.

Velocity methodology

After separating dwell and transition states, an additional analysis in the form of velocity versus position was used to elucidate enzyme behavior.

1. We separated dwells and transitions according to procedure described in the previous section.

2. We obtained and subtracted dwell averages to normalise data.

3. We determined position and velocity values. The relative position was obtained using the normalized cumulative angle data (of n values), while the velocity was obtained by taking the difference of consecutive points and dividing by the timestep (of n-1 values).

4. We binned the data according to angles and velocities thereby creating a 2-dimensional histogram. The data can be sorted using a hashmap or looping through predetermined angular position ranges averaging associated velocities at each range.

5. We plotted angular bin midpoints versus average rotation velocity (averaging is performed for each angular bin).

Robustness of velocity method compared to the angular position-based method in the transition range

Analysis of position versus time was found to be unreliable as the starting value majorly impacts the subsequent rate. Fig. S9 shows angular position versus time. As can be seen, the behavior varies significantly until approaching asymptotic behavior around 120° as it approaches the next dwell.

Rotational correction also proved valuable in enhancing the resolution of the average velocity as seen in Fig. S10. The comparability the subunits allowed greater resolution regarding transition behavior, as multiple subunits were able to be considered in tandem, effectively doubling or tripling the dataset.

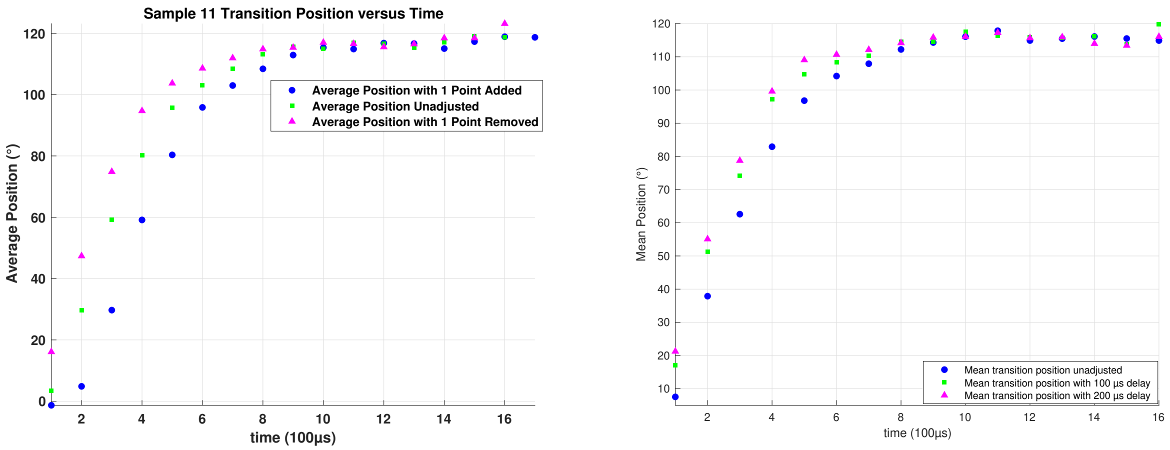

Fig. S9: Average Position versus Time with varied transition lag times for a) Sample 11 and b) Sample 13

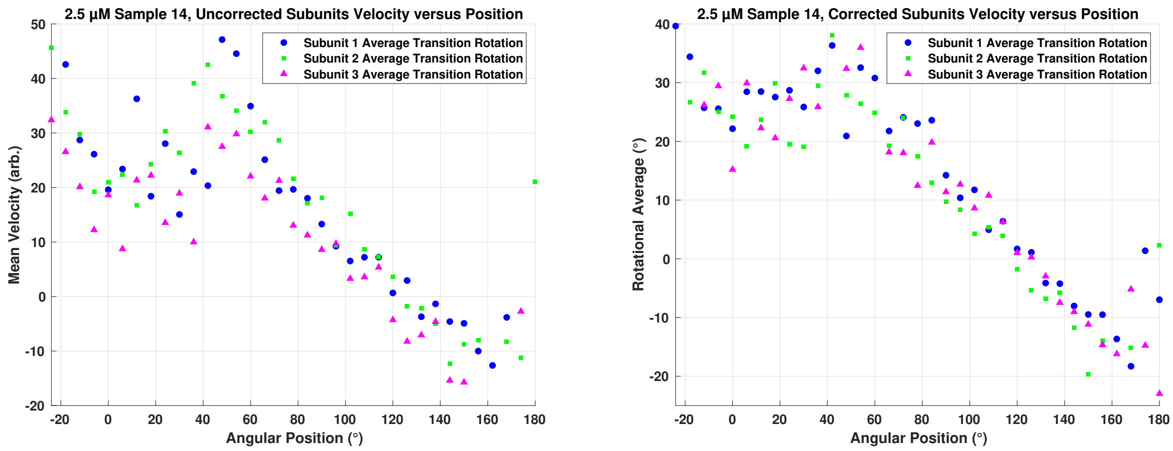

Fig. S10: Average Velocity versus Position plots for Sample 14 a) without rotation correction and b) with rotation correction

Contour plots of velocity vs angle can be seen in Fig. S11. The used samples show definitive states centered around 0° and 120°, with a plausible intermediate state between the initial and final states.

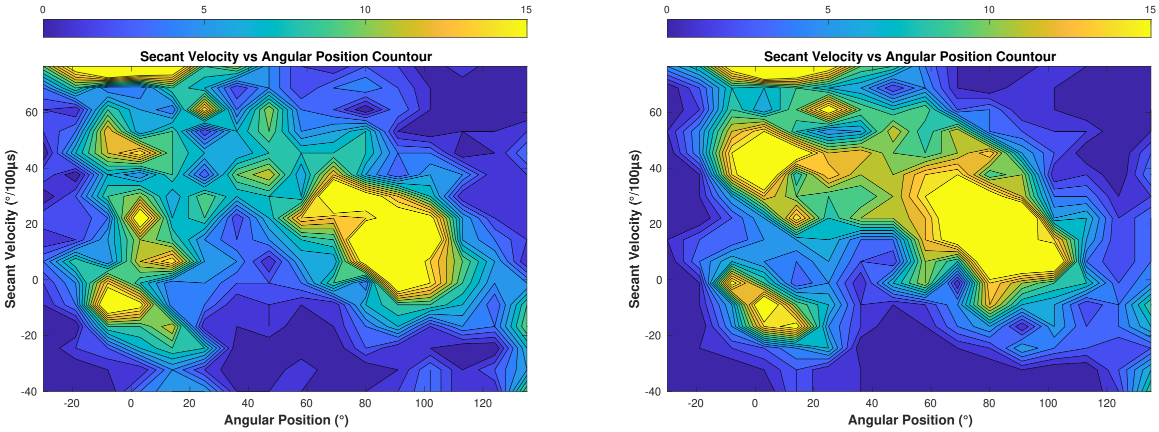

Fig. S11: Contour plots of velocity vs angle for a) Sample 13 and b) Sample 14

The velocity profile as a robust method circumventing the change-point problem

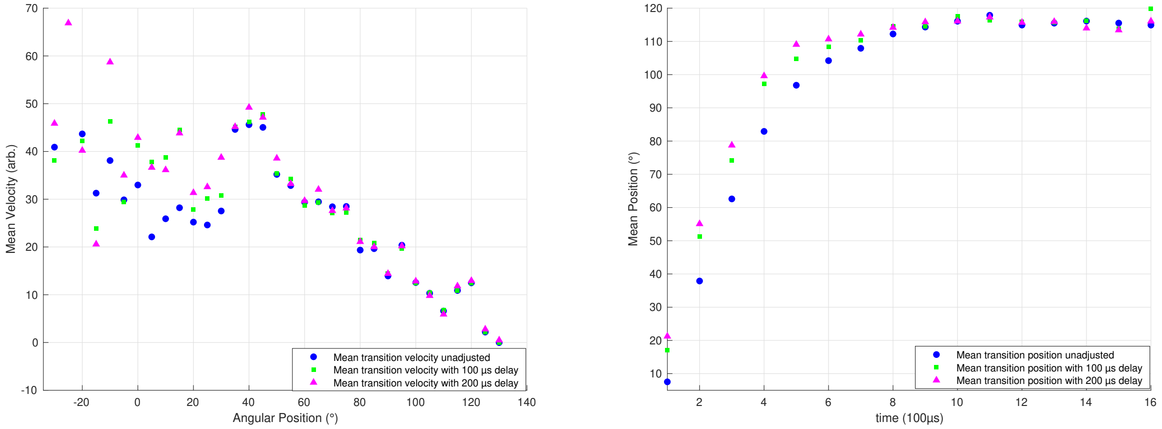

Fig. S12: Effect of changing the starting point of transitions on average velocity vs. angle (A) and average position vs. time (B) profiles. Three different transition starting points for the same trajectory (trajectory A).

The definition of the point where a dwell ends and a transition starts is an inversion problem similar to that of change-point analysis.[2] So, only approximate methods are used to define the change point, and in order to test the robustness of the velocity-based model, we performed the calculations by moving the change point both earlier and later than the initial definition. The latter had the benefit of possibly removing points that were falsely assigned to the transition, at the expense of losing data points from the transition. On Fig. S12 the effect of moving the change point is seen to be modest on the velocity profile. In all cases, the dip in the velocity – the hallmark of the short-lived state – persisted,l mso the velocity profile is robust with respect the the definition of the change point. This feature is desirable, and renders the velocity a reliable measure for extracting hidden states in the transition. In contrast, the average position was significantly altered, which means that the position is sensitive the the definition of the change point, so it is an unreliable measure for detecting hidden states.

Angle-dependent rate constant of the fast ADP release

For an arbitrary single-step transition,

$$k_{fi}(\theta) = k_{fi,0} \exp[a_{fi}(\theta - \theta_i)], \tag{1}$$

$$k_{bi}(\theta) = k_{bi,0} \exp[-a_{bi}(\theta - \theta_{i+1})]. \tag{2}$$

According to an elastic molecular group transfer model,[3] the angular coefficients are $a_{fi} = \beta\alpha_i\kappa_{ci}s_i$ and $a_{bi} = \beta(1-\alpha_i)\kappa_{ci}s_i$, where $s_i = (\theta_{i+1} - \theta_i)$ is the step size; κ_{ci} is the spring constant for the coupling between the mechanical rotation and the reaction coordinate for transition $i \to i+1$; $\beta = 1/k_BT$; and α_0 is the slope.[3–6]

From studies of controlled rotation experiments[7,8] the angular coefficient of ADP release is similar to that of ADP (or ATP) binding, so we use an experimentally determined value of $a_{f1} = 0.045$ deg^{-1}. The other two quantities, $k_{f1,0}$ and θ_1 are adjustable parameters to be

determined by fitting theory and experiment.

Theoretical calculations were performed both assuming an exponential angle dependence defined in Eqs. (1-1) using the value provided thereby. The results shown in Fig. S13 show that a best fit of the latter model cannot reproduce the dip and subsequent peak in the angular velocity vs. rotation angle in the experimental data. Instead, using an exponential angle dependence identical to that of ATP release or slow ADP release, the 2-state theory can be well fitted to the experimental angular velocity vs. angle data in the transition.

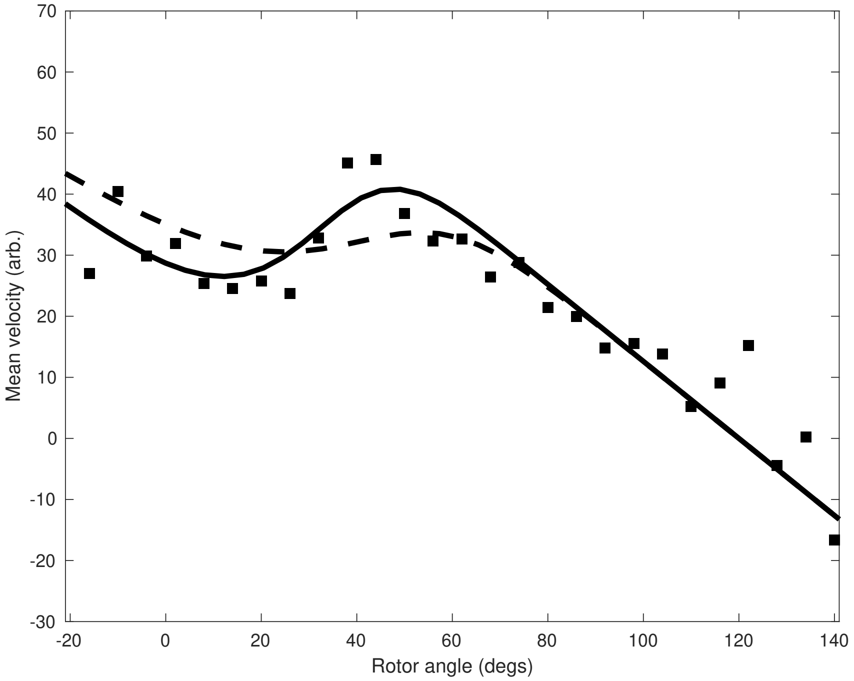

Fig. S13: Effect of angle dependence of the rate constant $k_{f,1}(\theta)$ for the fast ADP release. The solid line shows a best fit results of theoretical calculations assuming an exponential angle dependence. The dashed line show the best fit assuming no angular rate dependence. The exprimental data points are shown as squares.

Further assumptions in the multistate theory of rotation monitored by a nano-probe

In the model[9] we assume that the processes occur on two markedly different timescales: (1) the transfer of nucleotides (ATP or ADP) and smaller ions (inorganic phosphate P_i) in the binding channel take microseconds or less; and (2) the waiting time between these transitions, the video frame time of the apparatus and the motion of the probe itself are slower process that relax in micro- or milliseconds. The monitored θ is quasi-static during any particular transition,[3,8] which is the basis for a kinetics of angular position-dependent rate constants. Accordingly, a series of steps is described by a discrete 'chemical state' (or occupancy state) variable i. To each state i a dwell angle θ_i corresponds, that may be 'observable' in the imaging trajectories or hidden, if the state is too short-lived. During the transitions from i to $i+1$ transfer reactions occur with forward and back rate constants $k_{fi}(\theta)$ and $k_{bi}(\theta)$. When the system is in the reactant minimum θ fluctuates around the dwell angle θ_i; after a fast transition the system is found in the product state and θ relaxes and fluctuates about the next (product) dwell angle θ_{i+1}.

The i dependence of D and κ_r are assumed to be negligible since the former is determined by the size and shape of the probe, and the latter by linkage of the probe, the γ shaft and flexible elements of the stator ring. So ideally, none of these quantities change over the course of a trajectory. We note that the viscous drag of the probe is significantly affected by its interaction with the surface on which the F_1-ATPase was deposited. The interaction is likely angle-dependent, due to nanoscale features of the surface.

Angular velocity distributions

To calculate the angular velocity distributions we define the angle-time conditional probability distribution $\rho_{ii}^{pos}(\theta+\Delta\theta, t+\Delta t|\theta, t)$ that the system survives in state i at time $t+\Delta t$

and is found at angular position $\theta + \Delta\theta$, if at time t it was at θ. In this analysis Δt is the time step (frame time) of the imaging apparatus. $\rho_{ii}(\theta + \Delta\theta, t + \Delta t|\theta, t)$ satisfies Eq. **??** for a delta-function initial condition.[10] The solution is a Gaussian,[11] which has a θ-dependent peak, and is independent of t. A change of variable $\Delta\theta \to v$ from Eq. **??**, yields the conditional probability for an angular velocity $\rho^{vel}_{ii}(v, t + \Delta t|\theta, t) = \rho_{ii}(v|\theta)$ in a simplified time-suppressed notation,

$$\rho_{ii}(v|\theta) \cong \frac{1}{2\sqrt{\pi D/\Delta t}} \exp - \frac{v - v_{pi}(\theta)^2}{4D/\Delta t\,(1 - e^{-\tau/\Delta t})}. \tag{3}$$

Then a summation over all states yields

$$\rho(\Delta\theta/\Delta t|\theta) = \Delta t \sum_i \rho_{ii}(\Delta\theta|\theta) p_i(\theta). \tag{4}$$

Extracting the spring constant from dwells in the single-molecule trajectories

The fluctuations of the probe in single-molecule imaging can be detected accurately if the time step of the imaging apparatus Δt is smaller then the relaxation time τ.[1] We calculate the measured standard deviation σ_m of the angular position histogram compared to the true σ.

In single-molecule imaging, the position of the probe is detected during the imaging frame time Δt. For an imaging frame, when the system is in a given chemical state i (in a dwell), the measured angle $\theta_n = \overline{\theta}_{\Delta t}$, is a time average over the frame time,

$$\theta_n = \frac{1}{\Delta t} \int_{(n)\Delta t}^{(n+1)\Delta t} \theta dt. \tag{5}$$

Let's consider the auto-correlation function. The "true" time-time correlation function $C(t - t') = \langle \theta(t')\theta(t) \rangle$ is defined for $t' \le t$. For simplicity, the angles θ_1 and θ_m are shifted so that

the dwell mean angle is 0. The true-time correlation is then:

$$C(t - t') = \sigma^2 \exp[-(t - t')/\tau], \quad t' \leq t, \tag{6}$$

where $\sigma^2 = k_B T/\kappa$. The discrete correlation function for zero lag is,

$$C_0 = \frac{2}{\Delta t^2} \int_0^{\Delta t} dt \int_0^t dt' C(t - t'). \tag{7}$$

The factor of 2 is due to interchangeable between t and t' when both have same range from 0 to Δt. The variance is then calculated from equation 7,

$$\sigma_m^2 = C_0 = 2\frac{k_B T}{\kappa}\left[\frac{\tau}{\Delta t} - \frac{\tau^2}{\Delta t^2}(1 - e^{-\Delta t/\tau})\right] \tag{8}$$

Since $\sigma^2 \propto \kappa$, the variance allows relation between the true and measured value of the stiffness, κ and κ_m, respectively,

$$\kappa = 2\left[\frac{\tau}{\Delta t} - \frac{\tau^2}{\Delta t^2}(1 - e^{-\Delta t/\tau})\right]\kappa_m. \tag{9}$$

The measured angles will follow Gaussian distribution $p_m(\theta_m)$ whose width is scaled by a proportionality factor, according to Eq. 9. For a TBF1 trajectory with $\Delta t = 10\ \mu s$ analyzed previously,[?] an unbiased relaxation time of $\tau = 14\ \mu s$ was estimated from the correlation function. This value indicates that a significant correction factor of about 0.80 should be applied to the apparent $\kappa_m = 70$ pN.nm/rad^2, resulting in a spring constant of $\kappa_r = 56$ pN.nm/rad^2. In PDF1 with $\Delta t = 100\ \mu s$, an apparent elastic stiffness of $\tau_m = 27 pN.nm$ from Table **??** and relaxation time of about 35 μs the correction factor of 0.73 applies, indicating a true spring constant of $\tau = 20 pN \cdot nm$.

Fitting the angular velocity profile in the transition using parameters extracted from the dwell

For an accurate extraction of the lifetime and dwell angle of the short-lived state, it is desirable to have no fitting parameters in the model, except those describing the hidden state. Our strategy is to use the fluctuations from the dwells to extract the effective diffusion coefficient D of the probe and the effective spring constant $_r$ of the elements coupling the probe to the stator $\alpha_3\beta_3$ ring. The linear angular velocity-angle profiles predicted in the model in Eq. 4 and verified in experiment (Fig. S14) are used for the former.

The Gaussian angular histograms described in Eq. 9 and seen in experiment on Fig. S8 in the SI are used for the latter. Once these quantities are established, the 2-state model is used to calculate the velocities. Assuming as an initial condition that the system is in state 1 (the hidden state) and that the probability approaches zero at boundaries 2σ away from both $\theta_0 = 0$ and $\theta_2 = 120$, a two-state Eqs. 1-2 is solved numerically using a standard PDE solver in Matlab. The numerically calculated velocities then are compared with the angular velocity profile extracted from the points in the transitions (Fig. 4). The best fit then yields an estimate for the dwell angle θ_1 and lifetime $1/f_{f0,1}$ of the intermediate state. The procedure is performed separately for each trajectory, due to the (usually small) differences of probe size, attachment conformation and local environment.

Mean discrete angular velocity and its relation to the true mean angular velocity

We note that the mean angular velocity from Eq. 4 tends to the true mean angular velocity, $\langle v \rangle \to \langle v \rangle^{true}$, when the time step Δt tends to zero, i.e., if $\Delta t \ll \tau$. Nevertheless, the true mean angular velocity can be calculated for arbitrary Δt by the relation $\langle v^{true} \rangle = \langle v \rangle (\Delta t/\tau) \left(1 - e^{-\Delta t/\tau}\right)^{-1}$. If the time step of the apparatus Δt is significantly larger than τ

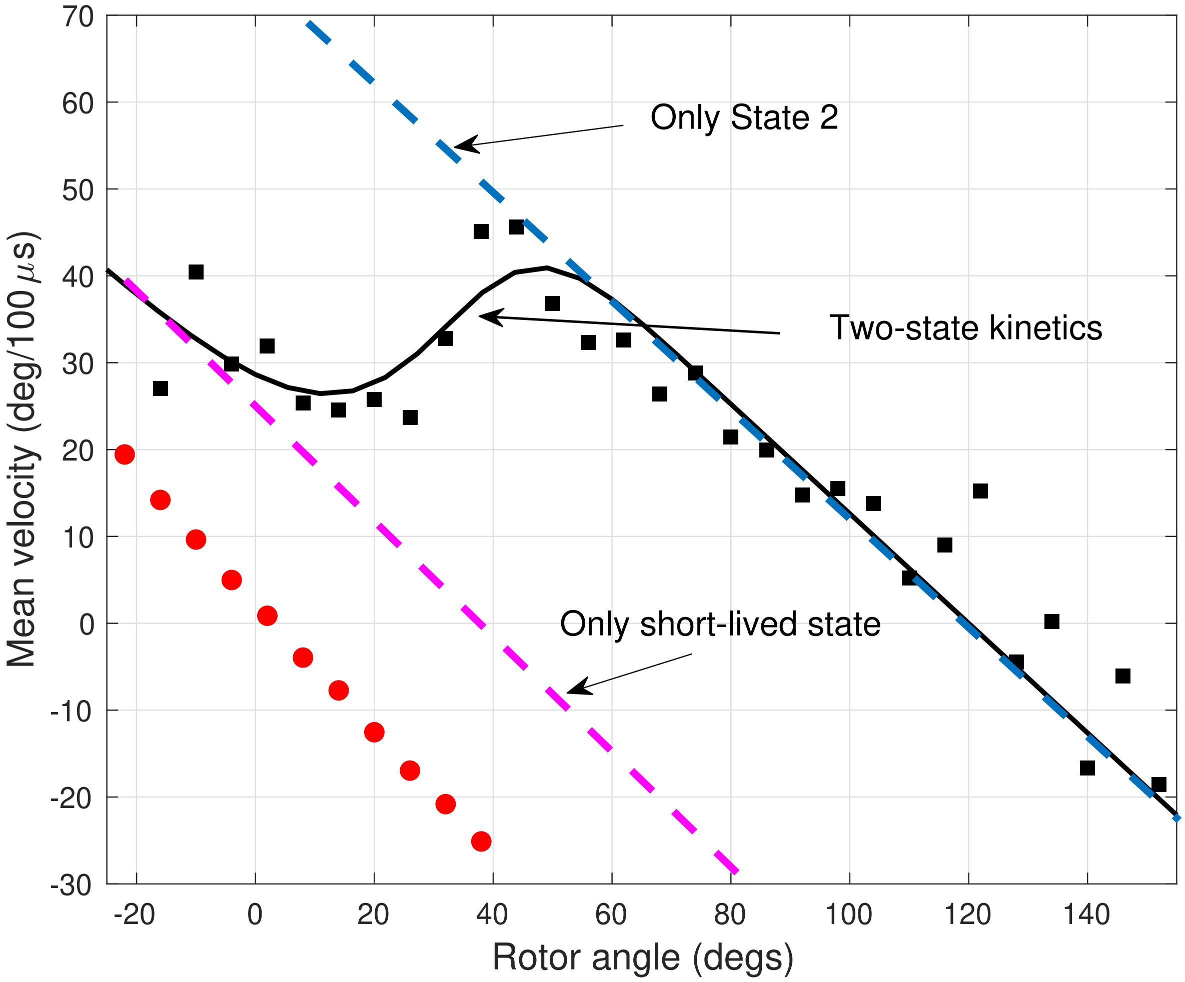

Fig. S14: Experimental and theoretical average angular velocity profile for a trajectory also used in Fig. 3 of the article. Black squares indicate the average angular velocity in the transitions between dwells, extracted from the single-molecule experiments, red circles show the linear velocity profile in the dwells preceding the transitions. The theoretical velocities assuming a two-state system with a short-lived intermediate state and a longer-live final state are shown as black solid lines. Dashed lines show theoretical calculations in the presence of only one of the states.

the 'correction factor' can become so large that the peak angular position (and the average jump) will have little connection with the 'true' average angular velocity. However, the latter can be still extracted from experiment if τ is know accurately, e.g., from the angular autocorrelation function in the dwell.

References

(1) Okuno, D.; Iino, R.; Noji, H. Stiffness of γ subunit of F_1-ATPase. *Eur. Biophys. J.* **2010**, *39*, 1589–1596.

(2) Watkins, L.; Yang, H. Detection of intensity change points in time-resolved single-molecule measurements. *J. Phys. Chem. B* **2005**, *109*, 617–628.

(3) Volkán-Kacsó, S.; Marcus, R. A. Theory for rates, equilibrium constants, and Brønsted slopes in F_1-ATPase single molecule imaging experiments. *Proc. Natl. Acad. Sci USA* **2015**, *112*, 14230–14235.

(4) Marcus, R. A. Theoretical Relations among Rate Constants, Barriers, and Brønted Slopes of Chemical Reactions. *J. Phys. Chem.* **1968**, *72*, 891–899.

(5) Szabo, A. Kinetics of hemoglobin and transition state theory. *Proc. Natl. Acad. Sci.* **1978**, *75*, 2108–2111.

(6) Schweins, T.; Warshel, A. Mechanistic Analysis of the Observed Linear Free Energy Relationships in p21ras and Related Systems. *Biochemistry* **1996**, *35*, 14232–14243.

(7) Adachi, K.; Oiwa, K.; Yoshida, M.; Nishizaka, T.; Kinosita, K., Jr. Controlled rotation of the F1-ATPase reveals differential and continuous binding changes for ATP synthesis. *Nat. Comm.* **2012**, *3*, 1022.

(8) Volkán-Kacsó, S.; Marcus, R. A. Theory of long binding events in single-

molecule–controlled rotation experiments on F_1-ATPase. *Proc. Natl. Acad. Sci USA* **2017**, *114*, 7272–7277.

(9) Volkán-Kacsó, S.; Le, L. Q.; Zhu, K.; Su, H.; Marcus, R. A. Method to extract multiple states in F1-ATPase rotation experiments from jump distributions. *Proceedings of the National Academy of Sciences* **2019**, *116*, 25456–25461.

(10) van Kampen, N. G. *Stochastic Processess in Physics and Chemistry*; North-Holland Publishing Company, 1981.

(11) Risken, H. *The Fokker-Planck Equation (2nd Edition)*; Springer, Berlin-Heidelberg, 1989.
